# *Tenebrio molitor* Larvae Inhibit Adipogenesis through AMPK and MAPKs Signaling in 3T3-L1 Adipocytes and Obesity in High-Fat Diet-Induced Obese Mice

**DOI:** 10.3390/ijms18030518

**Published:** 2017-02-28

**Authors:** Minchul Seo, Tae-Won Goo, Mi Yeon Chung, Minhee Baek, Jae-Sam Hwang, Mi-Ae Kim, Eun-Young Yun

**Affiliations:** 1 Department of Agricultural Biology, National Institute of Agricultural Sciences, RDA, Wanju-gun 55365, Korea; nansmc@hanmail.net (M.S.); shindelela@hanmail.net (M.Y.C.); minhee680@naver.com (M.B.); hwangjs@korea.kr (J.-S.H.); kimma@korea.kr (M.-A.K.); 2 Department of Biochemistry, School of Medicine, Dongguk University, Gyeongju 780-714, Korea; gootw@dongguk.ac.kr; 3 Graduate School of Integrated Bioindustry, Sejong University, Seoul 05006, Korea

**Keywords:** *Tenebrio molitor* larvae, diet-induced obesity, 3T3-L1, adipogenesis, hepatic steatosis

## Abstract

Despite the increasing interest in insect-based bioactive products, the biological activities of these products are rarely studied adequately. Larvae of *Tenebrio molitor*, the yellow mealworm, have been eaten as a traditional food and provide many health benefits. Therefore, we hypothesized that *T. molitor* larvae might influence adipogenesis and obesity-related disorders. In the present study, we investigated the anti-adipogenic and antiobesity effects of *T. molitor* larvae in vitro and in vivo. The lipid accumulation and triglyceride content in mature adipocytes was reduced significantly (up to 90%) upon exposure to an ethanol extract of *T. molitor* larvae, without a reduction in cell viability. Exposure also resulted in key adipogenic and lipogenic transcription factors. Additionally, in adipogenic differentiation medium the extract induced phosphorylation of adenosine monophosphate (AMP)-activated protein kinase and mitogen-activated protein kinases. Daily oral administration of *T. molitor* larvae powder to obese mice fed high-fat diet attenuated body weight gain. We also found that the powder efficiently reduced hepatic steatosis as well as aspartate and alanine transaminase enzyme levels in mice fed a high-fat diet. Our results suggest that *T. molitor* larvae extract has an antiobesity effect when administered as a food supplement and has potential as a therapeutic agent for obesity.

## 1. Introduction

The prevalence of obesity is now the leading public health problem worldwide. Obesity has an adverse effect on health, leading to increased comorbid metabolic and chronic diseases including type-2 diabetes mellitus, hypertension, coronary heart diseases, and cancer [1,2,3,4,5]. The ideal antiobesity drug should sustain weight loss through the reduction of energy intake or the increase of energy expenditure without adverse effects. Currently available antiobesity drugs successfully reduce body weight through the regulation of energy intake and expenditure but are often associated with serious side effects, such as headache, thirst, insomnia, constipation, and steatorrhea [6,7]. Therefore, there has been considerable interest recently in the development of antiobesity drug strategies that utilize natural bioactive substances and do not present side effects.

Many researchers have reported the presence of natural bioactive substances in a variety of sources such as plants, marine algae, and insects [1,8,9,10,11,12]. For example, plant extracts including polyphenols, resveratrol, curcumin, and proanthocyanidins, have become increasingly popular in the past decades for their anti-adipogenic properties [1,13,14]. It was also reported that herbal medicine was effective as potential therapy for fatty liver disease, diabetes, and metabolic syndrome [15]. Marine algae have also been shown to possess various biological activities such as antiasthmatic, antidiabetic, anticancer, and antioxidant effects [16,17,18]. However, insect-based bioactive products have rarely been studied, owing to a lack of scientific evidence regarding the safety and beneficial effects of insects despite their wide use as traditional foods or medicines in many countries. It has recently been suggested that insects will become the most popular source of effective bioactive products and dietary supplements for humans in the future [19]. Previous work has reported that insects could be useful sources for the development of medicine owing to their antidiabetic, antihepatofibrotic, anti-inflammatory, and anticancer properties [20,21]. 

*Tenebrio molitor* larvae (TML), also known as yellow mealworms, are eaten as a traditional food in many countries and provide many health benefits when taken as a dietary supplement, as they are rich source of nutrients including fats, minerals, and vitamins. In our previous work, we reported that oral administration of another insect, *Allomyrinal dichotoma*, inhibits the differentiation of adipocytes in vitro and in vivo [11,22]. Furthermore, intracerebroventriular (ICV) administration of an ethanol extract of *Allomyrinal dichotoma* larvae (ADL) reduces food intake and body weight via the regulation of appetite-related neuropeptides including neuropeptide Y (NPY), agouti-related protein (AgRP), and α-melanocyte-stimulating hormone (α-MSH) in high-fat diet (HFD)-fed mice [23]. 

Despite the increasing interest in insect-based bioactive products, the biological activities of these products have not been studied adequately. Thus, in the present study, we investigated the antiobesity effect of natural products extracted from TML in vitro and in vivo. Our results demonstrate TML has potential as a novel treatment option and can be developed as a therapeutic agent for obesity.

## 2. Results

### 2.1. Inhibitory Effect of Ethanol Extracts of Tenebrio molitor Larvae on Adipogenic Differentiation

**Figure 1:**
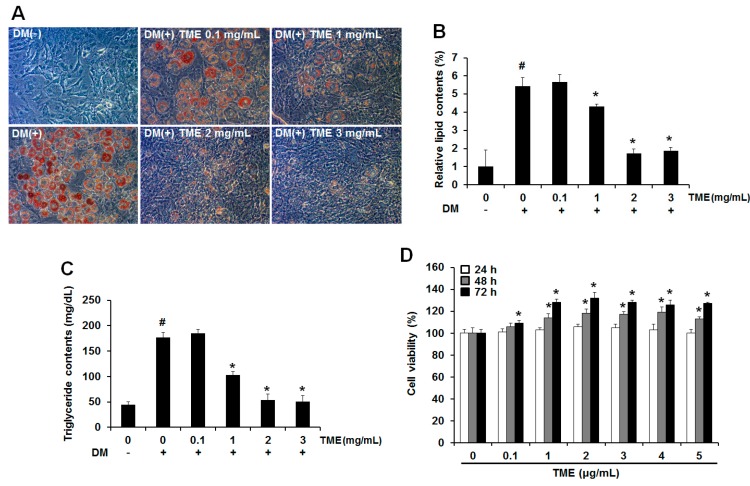
Antiadipogenic effect of ethanol extracts of *Tenebrio molitor* larvae (TME) on 3T3-L1 adipocytes. (**A**) Effect of TME on differentiation of 3T3-L1 adipocytes. 3T3-L1 adipocytes were cultured in adipocyte differentiation medium (DM) with or without treatment of TME. After 8 days of culture, cells were stained with Oil-Red O and photographed under a microscope (magnification: 40×). (**B**) Relative lipid contents and (**C**) Triglyceride (TG) content in different treatment groups. Results are presented as mean ± standard deviation (SD) of triplicate. ^#^ *p*-value of <0.05 indicates significant difference from non-differentiated control. * *p*-value of <0.05 indicates significant difference from differentiated control without TME. (**D**) Cytotoxicity of TME was determined using 3-(4,5-dimethylthiazol-2-yl)-5-(3-carboxymethoxyphenyl)-2-(4-sulfophenyl)-2H-tetrazolium (MTS) assay. Cells were seeded at a density of 2 × 10^5^ cells/mL in a 96 well-plate and treated with TME for 72 h. Results are presented as mean ± SD of triplicate. * *p*-value of <0.05 indicates significant difference from differentiated control without TME.

To evaluate the effect of ethanol extracts of *Tenebrio molitor* larvae (TME) on adipocytes, undifferentiated 3T3-L1 adipocytes were exposed to various concentrations of TME with differentiation medium (DM). The extract of *Ilex paraguariensis* (yerba mate), which has been reported to have antiobesity activity [24], was used as a positive control. Adipogenic differentiation of 3T3-L1 adipocytes was visualized on day 8 by Oil-Red O (Sigma, St. Louis, MO, USA) staining (Figure 1A). Mature adipocytes were identified and characterized based on the number of lipid droplets, which are not seen in undifferentiated cells. During adipogenic differentiation, the 3T3-L1 adipocytes, which were treated with TME, maintained their fibroblastic shape and contained fewer lipid droplets. Briefly, 1 and 2 mg/mL doses of TME significantly reduced the formation of lipid droplets to 20.58% ± 1.08% and 68.03% ± 0.98%, respectively, compared to that in mature adipocytes, indicating that TME could effectively inhibit adipocyte differentiation in 3T3-L1 adipocytes (Figure 1B). 

Intracellular triglyceride (TG) content was also measured on day 8 of adipogenic differentiation. Treatment with DM alone significantly increased TG content compared to undifferentiated cells. However, treatment with TME drastically reduced TG content by up to 41.14% ± 1.55% and 69.9% ± 2.23% at 1 and 2 mg/mL of TME, respectively (Figure 1C). In addition, no cytotoxic effects were observed at the concentrations of TME used in the current study (Figure 1D). Taken together, our results from the Oil Red O staining and TG assay demonstrated that TME inhibits the adipogenic differentiation of 3T3-L1 adipocytes.

### 2.2. Effect of TME on the Expression of Adipogenic Transcription Factors

**Figure 2:**
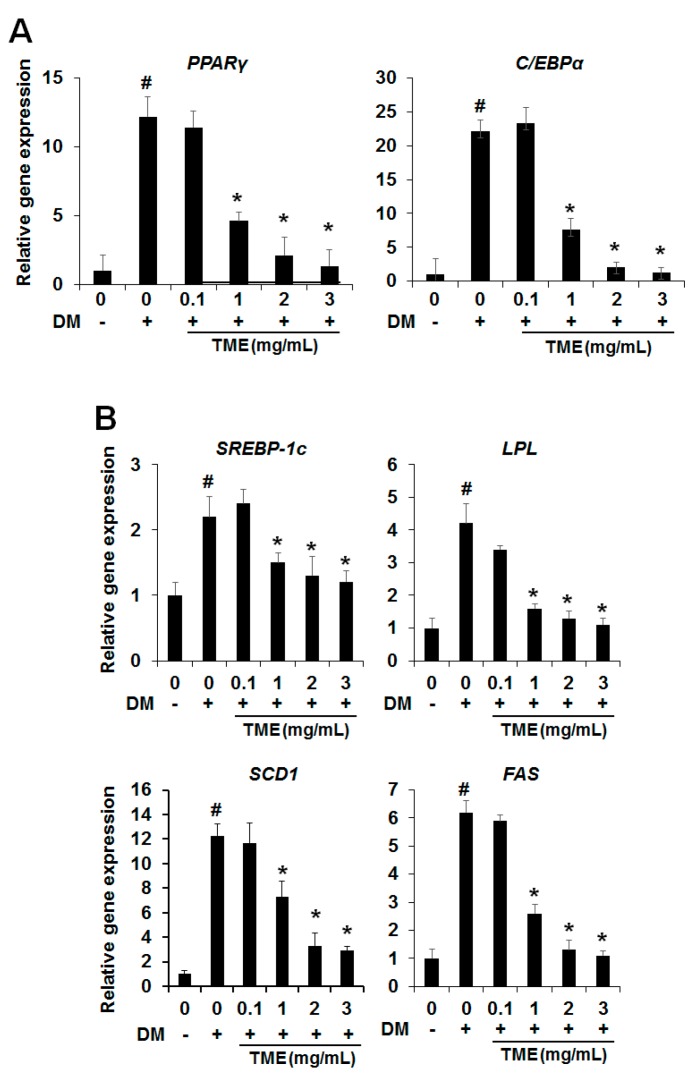
Effect of TME on gene expression of key adipogenic transcription factor- and adipocyte-specific markers. Cells were induced to differentiate into adipocytes in differentiation medium (DM) with or without TME and messenger RNA (mRNA) expression levels of adipogenic transcription factors (**A**); and adipocyte-specific markers (**B**) were measured using real-time polymerase chain reaction (PCR), respectively. Results are presented as mean ± SD of triplicate. ^#^ *p*-value of <0.05 indicates significant difference from non-differentiated control. * *p*-value of <0.05 indicates significant difference from differentiated control without TME. *PPARγ*: peroxisome proliferator-activated receptor gamma; *C/EBPα*: CCAAT/enhancer binding protein; *SREBP-1c*: sterol regulatory element binding transcription factor 1c; *LPL*: lipoprotein lipase; *SCD 1*: stearoyl-CoA desaturase-1; *FAS*: fatty acid synthase.

Adipogenic differentiation is regulated by the expression of related transcription factors, such as peroxisome proliferator-activated receptor gamma (PPARγ) and CCAAT/enhancer binding protein (C/EBPα), key transcription factors in adipogenesis. Therefore, we investigated whether TME influenced the messenger RNA (mRNA) expression levels of PPARγ and C/EBPα using real-time polymerase chain reaction (PCR) during adipogenic differentiation. As shown in Figure 2A, the mRNA expression levels of these genes were remarkably increased in the differentiated 3T3-L1 adipocytes. Meanwhile, the expression levels of PPARγ and C/EBPα were dose-dependently reduced in the differentiated 3T3-L1 adipocytes treated with various concentrations of TME compared with the untreated control cells.

### 2.3. Effect of TME on the Expression of Lipogenesis-Specific Genes

Next, we investigated whether the TME could affect the expression levels of lipogenesis-specific genes, such as sterol regulatory element binding transcription factor 1c (*SREBP-1c*), lipoprotein lipase (*LPL*), stearoyl-CoA desaturase-1 (*SCD1*), and fatty acid synthase (*FAS*)*.* These genes are key molecules in determining the phenotype of mature adipocytes. As shown in Figure 2, matured adipocytes had significantly increased mRNA expression levels for *SREBP-1c*, *LPL*, *SCD1*, and *FAS*. However, the increase in the mRNA levels of these genes in 3T3-L1 adipocytes was dose-dependently reduced on treatment with TME (Figure 2B). These results indicate that TME strongly suppressed lipid accumulation in 3T3-L1 adipocytes by reducing the expression levels of lipogenesis-specific genes.

### 2.4. Effect of TME on Phosphorylation of Adenosine Monophosphate (AMP)-Activated Protein Kinase (AMPK) and Mitogen-Activated Protein Kinases (MAPKs)

**Figure 3:**
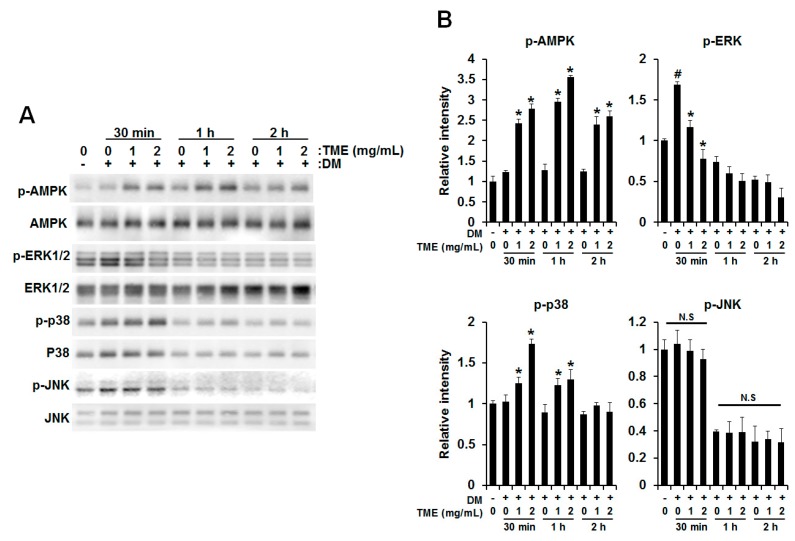
TME regulates phosphorylation of AMP-activated protein kinase (AMPK) and mitogen-activated protein kinases (MAPKs) during its inhibition of 3T3-L1 adipocyte differentiation. Cells were induced to differentiate into adipocytes in DM medium with or without TME for 30 min, 1 h, and 2 h; protein was extracted, and phosphorylation of AMPK, ERK, p38, and JNK was detected using western blot (**A**). Results of densitometric analysis of western blot are also shown (**B**). Experiment was performed three times using independently prepared cell lysates; representative blots are shown. # *p*-value of <0.05 indicates significant difference from non-differentiated control. * *p*-value of <0.05 indicates significant difference from differentiated control without TME. N.S: Not significant.

It is well-known that the AMP-activated protein kinase (AMPK) and mitogen-activated protein kinase (MAPK) signaling pathway regulates the expression of PPARγ and C/EBPα during adipogenesis in 3T3-L1 adipocytes [25,26,27]. Therefore, we examined whether TME influences the AMPK and MAPK signaling pathways. To evaluate the effect of TME, phosphorylation of AMPKα, p44/p42 MAPK (ERK), c-Jun N-terminal kinase (JNK), and p38 was determined. As shown in Figure 3, DM treatment led to increased phosphorylation of ERK within 30 min; however, TME treatment markedly decreased the level of DM-induced phosphorylation of ERK. In contrast, phosphorylation of AMPKα and p38 was dose-dependently increased by TME treatment. Unlike the above, DM-induced JNK phosphorylation was not changed by TME treatment.

### 2.5. Effect of TML on Body Weight and Adipose Tissue Weight

To evaluate the possibility that TML as a natural food supplement can regulate body and relative organ weight, mice were fed with either a normal-fat diet (ND) or a HFD for 6 weeks with or without TML. As a result, the final body weight gain for HFD mice was about 47% higher than ND mice. However, oral administration of TML significantly reduced body weight gain, by up to 19% (100 mg/kg of TML) and 25% (3000 mg/kg of TML) compared to mice fed only HFD (Table 1). After that, we investigated the weight of adipose tissues such as visceral fat and peripheral fat. Mice were sacrificed and the epididymal and abdominal-to-peripheral adipose tissues were then removed and weighed. As shown in Table 1, oral administration of TML (3000 mg/kg) reduced abdominal-to-peripheral adipose tissue and epididymal fat weight by up to 16% and 18%, respectively, compared to mice fed an HFD only. In the positive control, in mice fed an HFD with yerba mate (MT), abdominal-to-peripheral adipose tissue weight was approximately 44.4% lower than that of the HFD-only group, whereas the epididymal fat weight was 3.7% higher. This result is consistent with previous reports that a crude “yerba mate” does not decrease epididymal adipose tissue weight in rats significantly [28,29].

### 2.6. Effect of TML on Adipocyte Size and Hepatic Steatosis

**Figure 4:**
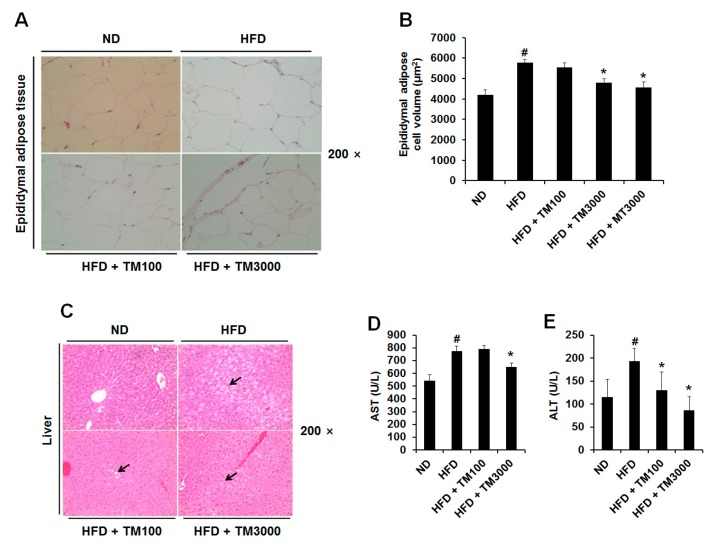
Effect of TML on histology of epididymal adipose and liver tissues of mice fed high-fat diet. (**A**) Histology of epididymal adipose tissues. (**B**) Epidiymal adipose cell volume was measured by IMT i-Solution Lite program (IMT i-solution Inc., Burnaby, BC, Canada). (**C**) Hepatic morphology. Arrows indicate macrovesicular lipid droplets in cytoplasm of hepatocytes. (**D**,**E**) Plasma transaminase activity. AST: aspartate transaminase; ALT: alanine transaminase. Results are presented as mean ± SD (*n* = 5). ^#^ *p*-value of <0.05 indicates significant difference from ND. * *p*-value of <0.05 indicates significant difference from HD. ND: normal-fat diet; HFD: high-fat diet; HFD + TM100: HFD and 100 mg/kg per day TML; HFD + TM3000: HFD diet and 3000 mg/kg per day TML; HFD + MT3000: HFD and 3000 mg/kg per day yerba mate (MT).

Next, we investigated the effect of TML on adipocyte size, as reducing adipocyte size is critical for the treatment of obesity and associated diseases [30,31]. As shown in Figure 4A,B, epididymal white adipose tissue size and volume in mice fed HFDs with TML significantly reduced compared with those in the mice fed an HFD only. Briefly, mean adipocyte volume in HFDs with 3000 mg/kg of TML was 16.9% lower than in HFD-only mice. Hepatic steatosis, the process of the abnormal retention of lipids, is the most common condition relating to the oversupply of lipids due to diabetes, obesity, and alcoholism. As shown in Figure 4C–E, TML significantly decreased the accumulation of hepatic lipid droplets along with plasma aspartate transaminase (AST) and alanine transaminase (ALT) levels.

### 2.7. Effect of TML on the Expression of Adipocyte Specific Genes In Vivo

**Figure 5:**
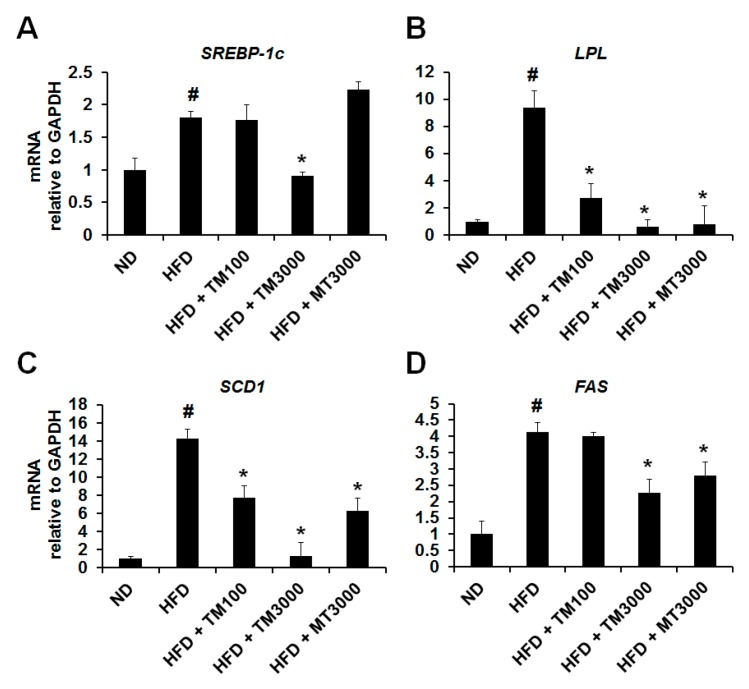
Effect of TML on expression of adipocyte specific markers in vivo. mRNA expression levels of *SREBP-1c* (**A**), *LPL* (**B**), *SCD1* (**C**), and *FAS* (**D**) in epididymal adipose tissue were analyzed by real-time PCR. Results are presented as mean ± SD of triplicate. ^#^ *p*-value of <0.05 indicates significant difference from ND. * *p*-value of <0.05 indicates significant difference from HFD. ND: normal-fat diet; HFD: high-fat diet; HFD + TM100: high-fat diet and 100 mg/kg per day TML; HFD + TM3000: HFD and 3000 mg/kg per day TML; HFD + MT3000: HFD and 3000 mg/kg per day yerba mate (MT). *GAPDH*: Glyceraldehyde 3-phosphate dehydrogenase.

In the present study, we confirmed that TME has an important role in the prevention of adipogenic differentiation and lipid accumulation in 3T3-L1 adipocytes via the regulation of adipogenesis and lipogenesis-related genes. In addition, oral administration of TML decreased adipose tissue weight and adipocyte size in HFD-fed obese mice. We therefore investigated the mRNA expression levels of lipogenesis related genes in epididymal adipose tissue. Compared with the ND group, the HFD-induced obese mice exhibited higher mRNA expression levels for *SREBP-1c*, *LPL*, *SCD1*, and *FAS* in the epididymal adipose tissue. However, oral administration of TML decreased mRNA expression levels of these genes compared with the HFD-only treatment (Figure 5). Here, we found that *SREBP-1c*, *LPL*, *SCD1*, and *FAS* expression in epididymal adipose tissue was effectively suppressed in the TML-treated group relative to the HFD-only group. 

## 3. Discussion

Beetles have been widely used in foods and oriental medicines to treat various diseases throughout the world. There has been an interest in developing effective bioactive products using beetles for health supplements or functional foods because of their nutrient components such as unsaturated fatty acids, proteins, vitamins, fiber, and minerals [11,32,33]. Recently, it was reported that *Protaetia brevitarsis* larvae (PBL) or *Allomyrinal dichotoma* larvae (ADL) have antioxidant, hepatoprotective, anti-neoplastic, and antiobesity properties [11,20,22,34]. In particular, ADL inhibited the differentiation of adipocytes through the downregulation of adipogenic or lipogenic transcription factors [11,22]. In addition, central administration of an ethanol extract of ADL reduced food intake and body weight through the regulation of appetite-related neuropeptides in high-fat diet (HFD)-fed mice [23]. In our previous study, we investigated the nutrient profiles of *Tenebrio molitor* larvae (TML) [33]. We found that TML contained higher levels of protein and nutrients compared with ADL, and speculated that like ADL, TML has a function as a good nutritional source as well as a natural antiobesity effect due to its nutrient profile.

First, we examined whether an ethanol extract of TML (TME) had anti-adipogenic effects. As shown in Figure 1, TME effectively reduced the formation of lipid droplets and the TG content at 1 mg/mL and 2 mg/mL of TME without cytotoxicity. The peroxisome proliferator-activated receptor gamma (PPARγ) and CCAAT/enhancer-binding protein alpha (C/EBPα) play pivotal roles in the regulation of preadipocyte differentiation [35]. Activation of these transcription factors results in terminal differentiation of adipocytes through the induction of lipogenesis related genes including sterol regulatory element binding transcription factor 1c (*SREBP-1c*), adipocyte fatty-acid binding protein (*aP2*), stearoyl-coenzyme desaturase-1 (*SCD1*), lipoprotein lipase (*LPL*), and fatty acid synthase (*FAS*) [36]. Therefore, we investigated the expression levels of adipogenic differentiation factors (PPARγ and C/EBPα) and lipogenesis-specific genes (*SREBP-1c*, *LPL*, *SCD1*, *FAS*) in vitro and in vivo. The mRNA expression levels of these genes were remarkably increased in the differentiated 3T3-L1 adipocytes and HFD-fed mice, but these genes were significantly decreased in both experiments when treated with various concentrations of TME and TML (Figure 2 and Figure 5).

AMP-activated protein kinase (AMPK) regulates cellular energy homeostasis and a number of biological pathways, such as carbohydrate and lipid metabolism. It has been reported that the phosphorylation of AMPK leads to β-oxidation by inactivating acetyl-CoA carboxylase (ACC) and upregulation of carnitine palmitoyltransferase I (CPT1) expression [37,38]. In particular, AMPK provides an upstream signal of PPARγ and inhibits differentiation of preadipocytes into adipocytes [25,26]. Furthermore, it is responsible for the inhibition of adipocyte differentiation by several compounds including apigenin, chitin, aspigenin, and epigallocatechin gallate [39,40]. In the present study, we found that TME increased the phosphorylation of AMPK during preadipocyte differentiation (Figure 3A). This result indicates that TME inhibits adipogenesis through the AMPK pathway. 

Mitogen activated protein kinases (MAPKs) such as ERK, p38, and JNK play a pivotal role in many essential cellular processes such as differentiation and proliferation [41]. Regarding the process of differentiation, the roles of MAPKs are extremely complex and depend on multiple parameters. Accumulating evidence suggested that the ERK has positive roles in adipocyte differentiation leading to the expression of the crucial adipogenic regulators, C/EBPα and PPARγ [30,42,43]. Consistent with this result, we found that the phosphorylation of ERK was increased by DM, which led to the increase of PPARγ and C/EBPα; however, TME markedly reduced DM-induced ERK phosphorylation in a dose-dependent manner (Figure 2 and Figure 3B). Contrary to ERK, the phosphorylation of p38 was increased by TME treatment (Figure 3B). Although previous works showed p38 has a positive role in adipocyte differentiation [44,45], many other reports have described the negative role of p38 in adipocyte differentiation [46,47,48]. These reports have indicated that TME reduces adipocyte differentiation by downregulation of ERK activity but upregulation of p38 activity.

Highly specialized adipocytes play a critical role in regulating energy balance and lipid metabolism [49]. Adipocytes are not simply storage depots for energy; they also secrete endocrine factors such as adipokines, cytokines, and growth factors when they reach at their limit capacity. Therefore, excess adipose tissue triggers obesity and associated metabolic diseases such as type-2 diabetes, cardiovascular disease, hypertension, and cancer by promoting inflammation and insulin resistance. We investigated the effect of TML on visceral fat mass and adipocyte size, since reducing visceral fat mass and adipocyte size appears to be critical for the treatment of obesity and its associated diseases [30,31]. Our results show that the visceral fat mass of mice fed HFD was reduced by oral administration of TML (Table 1). Furthermore, the size of adipocytes from epididymal white adipose tissue of mice fed HFD was also reduced (Figure 4A,B). Reducing visceral fat improved hepatic insulin action and reduced expression of excessive inflammatory cytokines, as visceral fat was considered as a key factor for obesity-associated diseases [50,51,52,53]. 

Abnormal lipid metabolism in obesity is considered a major driving force for dyslipidemia and hepatic steatosis [54]. In addition, non-alcoholic fatty liver disease and non-alcoholic steatohepatitis (NAFLD/NASH) is condition defined by excessive fat accumulation in the liver and displays liver cell injury and inflammation [55,56]. As shown in Figure 4C, increased hepatic steatosis in HFD was significantly decreased in TML-supplemented HFD-fed mice. The plasma levels of aspartate transaminase (AST) and alanine transaminase (ALT), reasonable sensitive indicators of liver damage or injury, as well as hepatic lipid droplet accumulation, were elevated in HFD-induced obesity [57]. In the present study, TML efficiently decreased hepatic lipid droplets accumulation along with plasma ALT and AST level. Accordingly, we suggest that TML may have therapeutic potential against not only HFD-induced metabolic disturbances such as dyslipidemia and hepatic steatosis but also NAFLD/NASH.

In conclusion, our most significant finding is that TML improved HFD-induced body weight gain, fat mass, adipose size, and hepatic steatosis. This data suggests the possibility to develop a therapeutic agent for obesity using TML due to its potent antiobesity effects both in vitro and in vivo. For the first time, this study has demonstrated the antiobesity activity of TML in vitro and in vivo. However, our result is not enough to fully understand and overcome obesity due to the complexity of the process and the possible crosstalk between the different pathways. Therefore, further studies are guaranteed to clarify which components of TME are responsible for these effects and the effect of appetite/satiety regulatory hormones such as ghrelin or leptin, and provide strong evidence for the antiobesity activity of TML.

## 4. Materials and Methods

### 4.1. Preparation of Tenebrio molitor Larvae Powder and Ethanol Extract

Freeze-dried *Tenebrio molitor* larvae (fdTML), sterilized (121 °C, 15 psi, 15 min) and ground to a powder, were provided by World Way Co. (Yeongi, Korea). The fdTML were mixed with ethanol (1 g of fdTML/10 mL of 70% ethanol) and incubated at room temperature 30 min after ultrasonication (250 J, 10 s, twice). After incubation, the supernatant was filtered and completely dried using a rotary evaporator. The dried ethanol extract of fdTML was dissolved in 20% dimethyl sulfoxide (DMSO) (TME). For oral administration of TML, fdTML powder was suspended in deionized water. The suspended TML solution was adjusted to concentrations of 100 mg/kg per day and 3000 mg/kg per day. A single oral dose was determined based on a previous report of a safety assessment of freeze-dried powdered *Tenebrio molitor* larvae [58]. The general components were measured using the official analytical methods of the Association of Official Analytical Chemists and the marker compound was measured using gas chromatography (GC) (GC-2010 Plus, Shimadzu, Japan) [24].

### 4.2. Cell Culture and Differentiation

Mouse 3T3-L1 pre-adipocytes were obtained from the American Type Culture Collection (Manassas, VA, USA) and incubated at 37 °C in a humidified 5% CO_2_ atmosphere in Dulbecco’s Modified Eagle Medium (DMEM) (Hyclone, Logan, UT, USA) containing 10% bovine calf serum (FCS) and 100 U/mL penicillin-streptomycin. Upon confluence (day 0), the media was replaced with adipogenic differentiation medium (DM) containing 10% fatal bovine serum (FBS), 10 μg/mL insulin, 0.5 mM 3-isobutyl-1-methylxanthine (IBMX), 1 μM dexamethasone (DEX), and various concentrations of TME. The culture was incubated at 37 °C, 5% CO_2_ for another 3 days. The media was then replaced with DMEM supplemented with 10% FBS and insulin (10 μg/mL) for 5 days. TME was added on day 0 during differentiation until the cells were harvested for the experiments.

### 4.3. In Vitro Cytotoxicity Assay

Mouse 3T3-L1 pre-adipocytes were seeded in a 96-well plate (1 × 10^4^ cells/well) and incubated at 37 °C with 5% CO_2_ with complete medium for 24 h. After 24 h, the medium was changed to complete medium supplemented with TME at various concentrations for 72 h. Cell viability was measured by a 3-(4,5-dimethylthiazol-2-yl)-5-(3-carboxymethoxyphenyl)-2-(4-sulfophenyl)-2*H*-tetrazolium (MTS; Sigma, St. Louis, MO, USA) assay. MTS solution was added to the plate and incubated for 4 h at 37 °C. The absorbance was then measured at 490 nm using a microplate reader (Beckman Coulter Co., Brea, CA, USA).

### 4.4. Oil Red O Staining

To investigate the effect of TME on lipid accumulation in 3T3-L1 pre-adipocytes, the cells were seeded in a 6-well plate (1 × 10^5^ cells/well) and differentiated in the presence of TME at various concentrations. Intracellular lipid accumulation was visualized by Oil Red O (Sigma, St. Louis, MO, USA) staining on day 8. The cells were washed with phosphate buffer saline (PBS), fixed with 10% formalin, washed with distilled water, and stained with Oil Red O solution (60% isopropanol in water) for 30 min. After rinsing three times with distilled water, the cells were photographed under a microscope (Leica CTR6000, Wetzlar, Germany). Then, Oil Red O was dissolved in 100% isopropanol and absorbance was measured by a microplate reader at 520 nm.

### 4.5. Triglyceride Content

To analyze the intracellular triglyceride (TG) content, the cells were washed with PBS, harvested by trypsinization and then suspended in a 1 ml solution containing 5% NP-40 in water. The samples were slowly heated to 80–100 °C in a water bath for 2–5 min or until the NP-40 became cloudy, and then cooled to room temperature. The heating was repeated once to ensure solubilizatiion of all TGs. The solution was then centrifuged for 2 min to remove any insoluble materials. TG content was determined using a commercial TG assay kit according to the manufacturer’s protocol (Wako Chemicals Inc., Osaka, Japan). The protein concentration was determined using the bicinchoninic acid (BCA) Protein Assay kit (Thermo Scientific, Waltham, MA, USA). To measure the serum TG content, serum was separated from blood samples by centrifugation at 3000× *g* for 15 min. The serum TG concentration was evaluated spectrophotometrically, using commercially available diagnostic kits supplied by Asan Pharmaceutical (Seoul, Korea).

### 4.6. Reverse Transcription-PCR

Total RNA was extracted from epididymal adipose tissue or cells using Trizol reagent (Invitrogen, Carlsbad, CA, USA), according to the manufacturer’s instructions. The concentration or purity was measured using an Ultraviolet (UV) spectrophotometer. Complementary DNA (cDNA) was synthesized with the High Capacity cDNA Reverse Transcription Kit (Applied Biosystems, Carlsbad, CA, USA) using 1 μg of total RNA. Real-time PCR was performed with the specific primer set in Table S1. Glyceraldehyde 3-phosphate dehydrogenase (*GAPDH*) was used as an internal control.

### 4.7. Protein Extraction and Western Blot

Tissue or cells were lysed in RIPA lysis buffer (50 mM Tris-HCl, pH 8.0, 150 mM NaCl, 0.02% sodium azide, 0.1% sodium dodecyl sulfate (SDS), 1% NP-40, 0.5% sodium deoxycholate, and 1 mM phenylmethylsulfonyl fluoride). Protein concentrations of cell lysates were measured using the BCA Protein Assay kit. Equal amounts of protein were separated by 10%–12% SDS-polyacrylamide gel electrophoresis (PAGE) and transferred to polyvinylidene difluoride PVDF membranes (Bio-rad, Hercules, CA, USA). Membranes were blocked with 5% skim milk and sequentially incubated with the following primary antibodies followed by detection using an enhanced chemiluminescence (ECL) detection kit (Invitrogen, Waltham, MA, USA) with ChemiDoc imaging systems (Alpha Innotech Corp., Santa Clara, CA, USA): rabbit polyclonal anti-phospho-AMPKα antibody (1:1000; Cell Signaling Technology, Danvers, MA, USA); rabbit polyclonal anti- AMPKα antibody (1:1000; Cell Signaling Technology); rabbit polyclonal anti-phospho-p44/p42 MAPK (ERK1/2) antibody (1:1000; Cell Signaling Technology); rabbit polyclonal anti-p44/p42 MAPK (ERK1/2) antibody (1:1000; Cell Signaling Technology); rabbit polyclonal anti-phospho-p38 MAPK antibody (1:1000; Cell Signaling Technology); rabbit polyclonal anti-p38 MAPK antibody (1:1000; Cell Signaling Technology); rabbit polyclonal anti-phospho-JNK antibody (1:1000; Cell Signaling Technology); rabbit polyclonal anti-JNK antibody (1:1000; Cell Signaling Technology); β-actin antibody (1:1000; Santa Cruz Biotechnology, Santa Cruz, CA, USA), and horseradish peroxidase (HRP)-conjugated secondary antibody (1:10,000; Promega, Maddison, WI, USA).

### 4.8. Animals and Diets

Male BALB/c mice (5 weeks of age) were obtained from Central Lab Animal, Inc. (Seoul, Korea). Mice were allowed free access to a standard chow diet and water for 1 week. To generate diet-induced obesity (DIO), 6-week-old mice were randomly assigned to one of five treatment conditions: normal-fat diet (ND) (*n* = 7, 10% fat, D12450B; Research Diets, New Brunswick, NJ, USA); HFD (*n* = 7, 60% fat, D12492; Research Diets); HFD with 100 mg/kg of TML (*n* = 7); HFD with 3000 mg/kg of TML (*n* = 7); and HFD with 3000 mg/kg of yerba mate (MT) (*n* = 7). The mice were fed with their assigned diets for 6 weeks. TML and MT were administrated daily via oral gavage using disposable mouse feeding needles (Scientific Hub Services Pte Ltd., Singapore). The mice were placed in a controlled temperature room (23 °C) with a 12 h light/12 h dark cycle with free access to food and water. Body weight was measured weekly and weight gain was calculated as the difference between initial and final body weights, divided by initial body weight. At the end of the experimental period, the mice were fasted for 12 h prior to sacrifice. Blood samples were centrifuged at 3000 rpm for 15 min at 4 °C and plasma levels of AST and ALT were measured. After cervical spine dislocation, abdominal-to-peripheral adipose tissue, epididymal adipose tissue, and liver were dissected. All procedures followed the Principles of Laboratory Animal Care (NIH, USA) and were approved by the National Academy of Agricultural Science (NAAS-1403).

### 4.9. Histological Analysis

The liver and epididymal adipose tissues were fixed with 10% formalin for 48h. Fixed tissues were dehydrated in ethanol graded from 75% to 100% and embedded in paraffin. The embedded tissues were sectioned (8 μm), stained with hematoxylin and eosin (H&E) and then photographed by light microscopy (Leica CTR6000, Hesse, Germany). The epididymal adipose cell volume was estimated using the IMT i-Solution Lite (version 8.0, IMT i-Solution Inc., New York, NY, USA)

### 4.10. Statistical Analysis

All data was presented as the means ± SDs. Two groups were compared with Student’s *t*-test. Comparisons between three or more groups were analyzed using one-way ANOVA with Dunnett experiments. SPSS version 18.0 K (SPSS Inc., Chicago, IL, USA) was used for the analysis, and *p*-value differences of <0.05 were considered statistically significant.

## Figures and Tables

**Table 1:** Effect of oral administration of *Tenebrio molitor* larvae (TML) on body weight gain and fat weight in mice fed a high-fat diet.

Group	Body Weight (g)		Weight Gain (g)	Relative Fat Weight	
	Initial	Final		Peripheral	Epididymal
ND	33.15 ± 0.66	45.58 ± 1.61	0.37 ± 0.13	2.99 ± 0.61	0.177 ± 0.01
HFD	32.45 ± 0.77	57.97 ± 6.62	0.78 ± 0.29	7.72 ± 0.52	0.178 ± 0.03
HFD + TM100	31.98 ± 1.15	52.23 ± 4.77	0.63 ± 0.30	7.21 ± 0.53	0.161 ± 0.03
HFD + TM3000	32.78 ± 0.27	51.82 ± 3.99	0.58 ± 0.28	6.45 ± 0.62	0.146 ± 0.02
HFD + MT3000	32.10 ± 1.33	45.72 ± 4.74	0.42 ± 0.18	4.29 ± 0.31	0.185 ± 0.02

ND: normal-fat diet; HFD: high-fat diet; HFD + TM100: high-fat diet and 100 mg/kg per day TML; HFD + TM3000: high-fat diet and 3000 mg/kg per day TML; HFD + MT3000: high-fat diet and 3000 mg/kg per day yerba mate (MT). Weight gain (g) = (final body weight − initial body weight)/initial body weight. Relative fat weight = (fat weight/body weight) × 100. The results are means ± SDs (*n* = 7 per group).

## Supplementary Materials

The following are available online at http://www.mdpi.com/1422-0067/18/3/518/s1.
