# Supplementary material for: Tenebrio molitor Larvae Inhibit Adipogenesis through AMPK and MAPKs Signaling in 3T3-L1 Adipocytes and Obesity in High-Fat Diet-Induced Obese Mice
Source: Int J Mol Sci. 2017 Feb 28;18(3):518. doi: 10.3390/ijms18030518 (PMC5372534; doi:10.3390/ijms18030518)
Supplement: Supplementary file 1 [file ijms-18-00518-s001.pdf]

Supplementary Materials: *Tenebrio molitor* Larvae Inhibit Adipogenesis through AMPK and MAPKs Signaling in 3T3-L1 Adipocytes and Obesity in High-Fat Diet-Induced Obese Mice

Minchul Seo, Tae-Won Goo, Mi Yeon Chung, Minhee Baek, Jae-Sam Hwang, Mi-Ae Kim and Eun-Young Yun

Table S1. Primer sequences for amplification of genes involved in adipose metabolism.

Gene Name	Gene ID	Forward Primer	Reverse Primer
GAPDH	NM_008084	AAGAAGGTGGTGAAGCAGGCATC	CGAAGGTGGAAGAGTGGGAGTTG
PPAR γ	NM_011146	TTCAGCTCTGGGATGACCTT	CGAAGTTGGTGGGCCAGAAT
C/EBP α	NM_007678	GTGTGCACGTCTATGCTAAACCA	GCCGTTAGTGAAGAGTCTCAGTTTG
FAS	NM_007988	TTGCTGGCACTACAGAATGC	AACAGCCTCAGAGCGACAAT
SCD1	NM_009127	CATCGCCTGCTCTACCCTTT	GAACTGCGCTTGGAAACCTG
SREBP-1c	NM_011480	ATCGCAAACAAGCTGACCTG	AGATCCAGGTTTGAGGTGGG
LPL	NM_008509	TCCAAGGAAGCCTTTGAGAA	CCATCCTCAGTCCCAGAAAA
